# Genetic imprints of grafting in wild iron walnut populations in southwestern China

**DOI:** 10.1186/s12870-023-04428-z

**Published:** 2023-09-13

**Authors:** Jie Liu, Ephie A. Magige, Peng-Zhen Fan, Moses C. Wambulwa, Ya-Huang Luo, Hai-Ling Qi, Lian-Ming Gao, Richard I. Milne

**Affiliations:** 1 CAS Key Laboratory for Plant Diversity and Biogeography of East Asia, Kunming Institute of Botany, Chinese Academy of Sciences, Kunming, Yunnan 650201 China; 2 Germplasm of Bank of Wild Species, Kunming Institute of Botany, Chinese Academy of Sciences, Kunming, Yunnan 650201 China; 3 University of the Chinese Academy of Sciences, Beijing, 100049 China; 4 Department of Life Sciences, School of Science and Computing, South Eastern Kenya University, Kitui, 170-90200 Kenya; 5 Lijiang Forest Biodiversity National Observation and Research Station, Kunming Institute of Botany, Chinese Academy of Sciences, Lijiang, Yunnan 674100 China; 6 School of Ecology and Environmental Science, Yunnan University, Kunming, Yunnan 650091 China; 7 Institute of Molecular Plant Sciences, School of Biological Sciences, University of Edinburgh, Edinburgh, UK

**Keywords:** Conservation, Genetic diversity, Genetic erosion, Grafting, Iron walnut, *Juglans sigillata*, Microsatellite

## Abstract

**Background:**

Anthropogenic activities are causing unprecedented loss of genetic diversity in many species. However, the effects on genetic diversity from large-scale grafting onto wild plants of crop species are largely undetermined. Iron walnut (*Juglans sigillata* Dode) is a deciduous nut tree crop endemic to southwestern China with a long history of cultivation. Due to the rapid expansion of the walnut industry, many natural populations are now being replaced by cultivars grafted onto wild rootstocks. However, little is known about the potential genetic consequences of such action on natural populations.

**Results:**

We sampled the scion and the rootstock from each of 149 grafted individuals within nine wild populations of *J. sigillata* from Yunnan Province which is the center of walnut diversity and cultivation in China, and examined their genetic diversity and population structure using 31 microsatellite loci. Scions had lower genetic diversity than rootstocks, and this pattern was repeated in seven of the nine examined populations. Among those seven populations, AMOVA and clustering analyses showed a clear genetic separation between all rootstocks and all scions. However, the two remaining populations, both from northern Yunnan, showed genetic similarity between scions and rootstocks, possibly indicating that wild populations here are derived from feralized local cultivars. Moreover, our data indicated probable crop-to-wild gene flow between scions and rootstocks, across all populations.

**Conclusions:**

Our results indicate that large-scale grafting has been causing genetic diversity erosion and genetic structure breakdown in the wild material of *J. sigillata* within Yunnan. To mitigate these effects, we caution against the overuse of grafting in wild populations of iron walnut and other crop species and recommend the preservation of natural genotypes through in situ  and ex situ conservation.

**Supplementary Information:**

The online version contains supplementary material available at 10.1186/s12870-023-04428-z.

## Background

As a fundamental dimension of biodiversity, genetic diversity represents evolutionary potential, determining long-term maintenance of species [1, 2]. In plants, the breeding system and mode of proliferation contribute significantly to shaping the genetic diversity and population structure [3, 4]. Sexual reproduction based on seed propagation, which usually occurs in natural populations, serves to preserve the evolutionary potential of species since the genetically variable offspring can survive and adapt to varying environmental conditions [5–7]. Natural populations of outcrossing species are usually characterized by high within-population diversity and low among-population differentiation [8]. Conversely, clonal propagation, being the commonest type of asexual reproduction [9], has been associated with reduced genetic diversity, especially when it occurs intensively, and can bring about a subsequent decrease in the levels of genetic differentiation within populations, or groups thereof derived from the same source [10–12]. This can also lead to genetic sub-structuring among populations [11].

For cultivated plants, clonal propagation, including the regeneration of whole plants via cuttings or grafts, allows for phenotype uniformity, with the desired agronomic traits faithfully passed on to subsequent generations [13]. This has benefits, including increased yield and quality of produce, plus guarantees of efficient nutrient acquisition, accumulation of biomass, and reduced mortality risk when a strong cultivar genotype is selected [9, 12]. This might explain the widespread shift from traditional seed-based breeding to vegetative propagation in many cultivated plant species. However, the resultant clonality often leads to reduced sexual recombination, or none at all [14]. This, coupled with the intensive cultivation of genetically identical cultivars, is likely to reduce the genetic variability within a species, in turn decreasing the species’ adaptability and resilience in the wake of biotic or abiotic stressors. Therefore, it is important to investigate the long-term effects of replacing seed-propagated individuals with grafted cultivars, particularly in long lived tree crops.

Grafting is the fusion of two plant parts so that vascular function can establish between them [15], and the resulting united individual can normally grow like a single plant [16]. Indeed, this has been a pivotal technology through much of human history and is now widely applied in agriculture, horticulture and biology [16] (e.g., rose, cucumber, eggplant, tomato, watermelon, apple, citrus, grape, and pear [17–19]). When, where and how fruit tree grafting began is still debated [16, 19], with the earliest reliable written evidence of grafting dating to 412 BCE [16], although the technique might have been used in ancient Mesopotamia around 1800 BCE [20]. Grafting in walnut is much more difficult than in many cultivated taxa because it is impeded by poor callus formation, and hence requires greater precision in terms of time, temperature, humidity, selection, and the expertise to handle scions and stocks. Therefore, traditional propagation of walnut remained through seed for a long time, but these challenges have gradually been resolved by introducing more developed grafting methods such as hot callus, epicotyl grafting, and water nutrient solution systems [21, 22]. Once grafting became feasible, market pressures hastened the replacement of natural seed-propagated walnut trees with grafted cultivars, creating great challenges for walnut resources conservation.

**Fig. 1:**
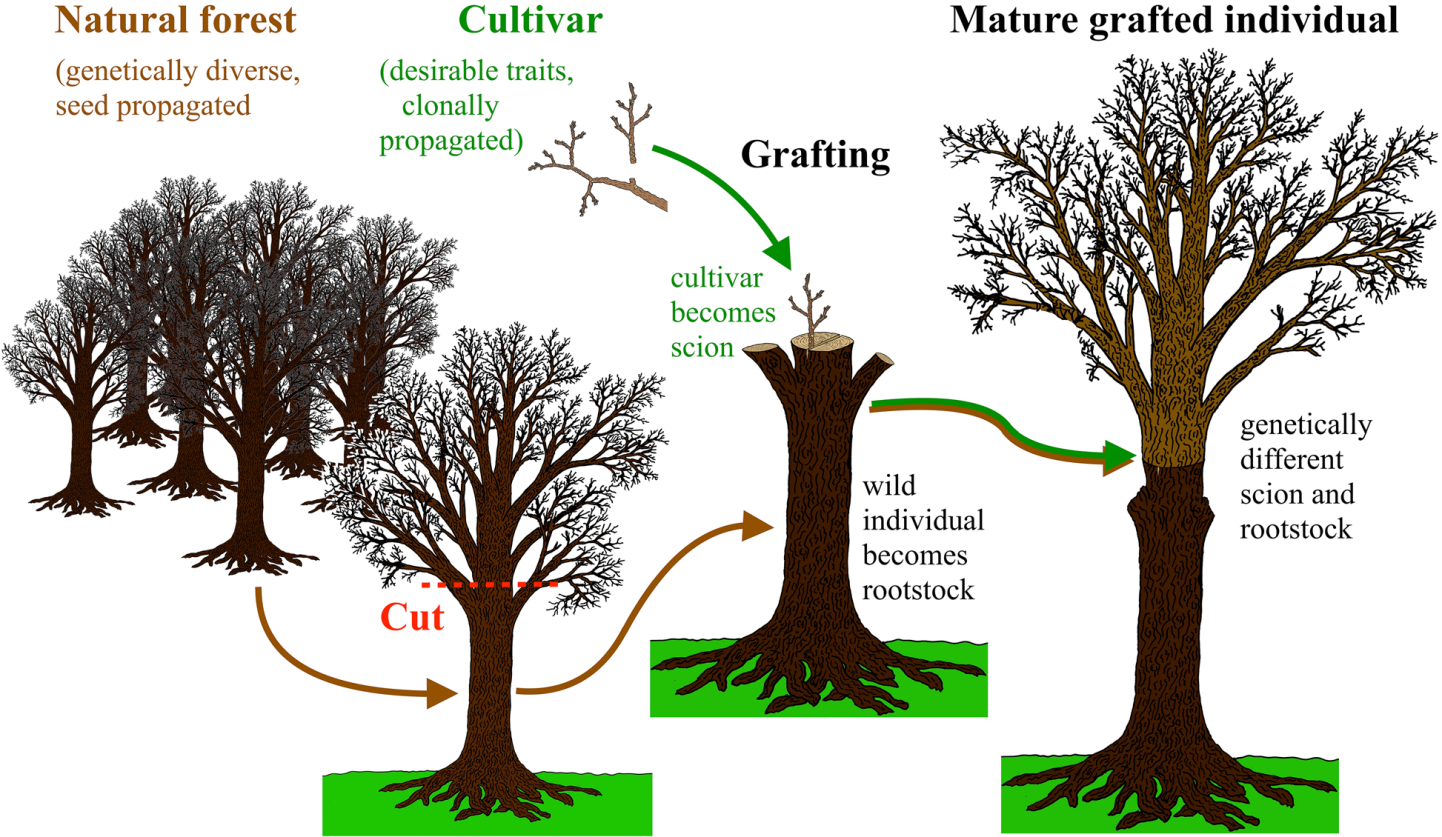
A schematic illustration of grafting in iron walnut from the natural forest to the mature grafted individual. The natural forest reproduces sexually through seed propagation, while the cultivar is propagated asexually by cloning. The resultant tree will have different genotypes between the scion and rootstock. The figure was created by Drs. Richard Milne and Jie Liu

**Fig. 2:**
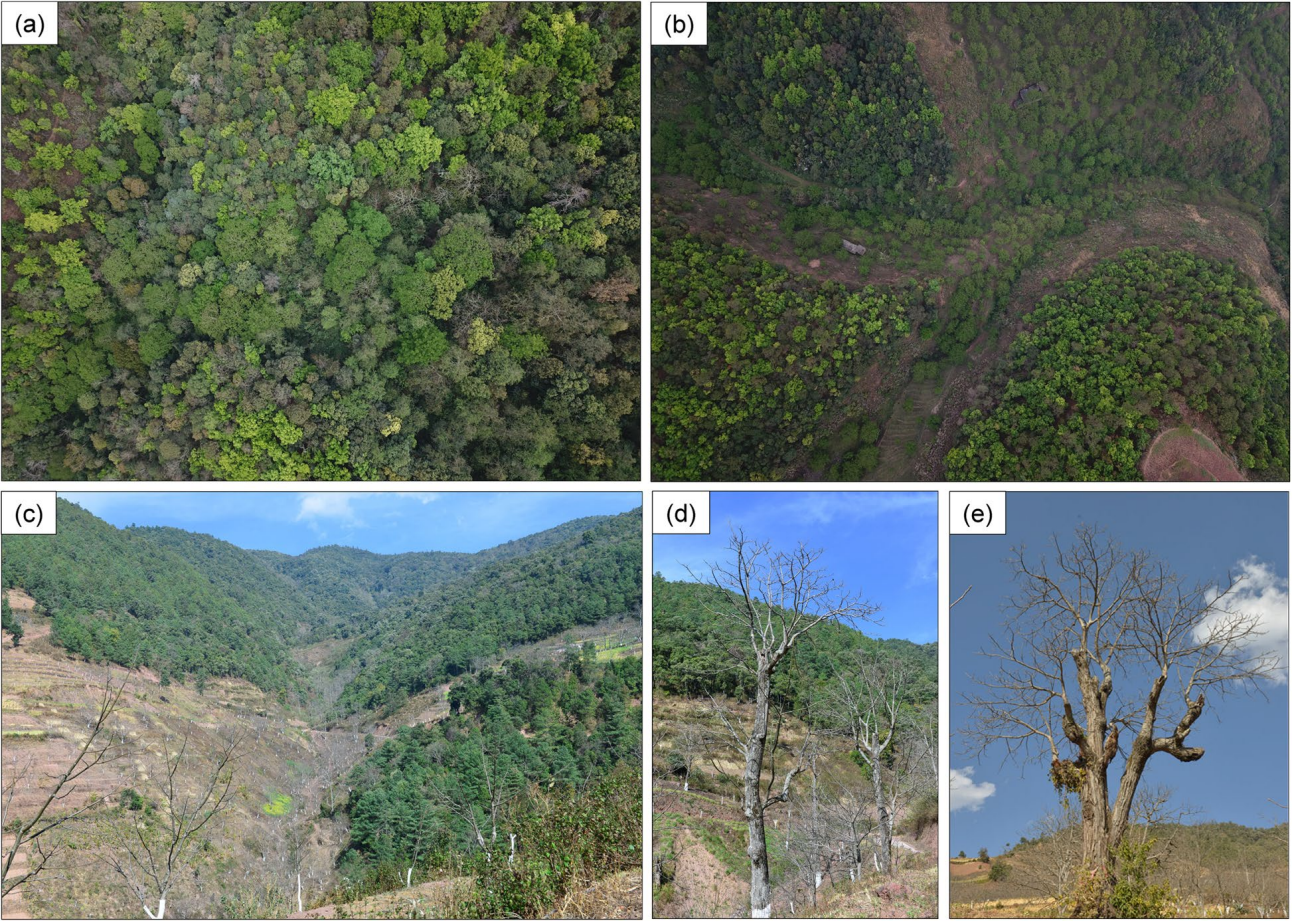
Photographs of *Juglans sigillata* forest and trees in Goujie Xiang (耈街乡), Changning County (昌宁县), Yunnan Province. **a** the natural forest of *J. sigillata* in the valley near the Longtan village (龙潭组); **b**-**c** natural iron walnut forest disturbed by recent artificial grafting and plantation; **d**-**e** grafted individuals of natural iron walnut trees, with branches and trunks representing scions and rootstocks, respectively. Photos courtesy of Dr. Jie Liu and Mr. Tao Liu

Yunnan Province is well known for its high biodiversity [23], and is the most important center of walnut genetic diversity, plantation, and production in China [24]. The common, Persian or English walnut (*J. regia* L.) is mainly cultivated in the northwestern parts, whereas both wild and cultivated forms of iron walnut (*J. sigillata* Dode) are widely distributed in subtropical region of Yunnan. Iron walnuts are highly valued for their nutritious nuts and high-quality timber, as well as its religious and cultural significance [25, 26]. Wild material of this species shows more resistance to pathogens and pests than do cultivars such as ‘Dapao’ [24]. Historically, cultivated populations were mainly maintained by grafting onto rootstocks grown from wild iron walnut seed. However, grafting has recently been widely applied to natural iron walnut forests (Figs. 1 and 2), as a consequence of the rapid expansion of the walnut industry over the past three decades. Consequently, sexual reproduction is increasingly being replaced by artificial asexual reproduction. Such wild populations now contain both grafted and naturally seed-propagated individuals, and can hence act as an ideal model to test the population genetic effects of grafting. Thus far, to the best of our knowledge, comparative analysis of genetic diversity of the scion and rootstock components has only been undertaken for Mediterranean old olives [27], with rootstocks showing higher genetic diversity than scions. However, that study [27] involved only cultivated trees, and we know of no other study examining the genetic impact of grafting within natural populations. Furthermore, little attention has been paid to the effect of grafting on the genetic diversity and population structure of wild stands of walnuts.

Simple sequence repeats (SSRs) are effective molecular markers, which have been widely and successfully used to infer the level of genetic diversity and population structure in different fruit trees including walnut (e.g. [28–30]). Therefore, to examine the genetic impacts of grafting on natural populations of walnuts, and more generally, we used 31 nuclear SSRs to examine nine populations of scions and rootstocks each, to investigate the effect of grafting on the genetic diversity and population structure of wild iron walnut trees in Yunnan. Our results will contribute towards data-driven breeding and conservation of walnut in Yunnan and beyond.

## Results

### Genetic diversity

A total of 298 samples (Table 1) were successfully screened using 31 microsatellite loci (Table S1). The set of SSR loci used in the study showed a high level of polymorphism (Table S2), minimum stuttering and allele drop-out, low null allele frequencies, and only a few population-locus combinations deviated significantly from Hardy–Weinberg equilibrium (HWE), as per our previous studies [28, 31]. Several clonemates were identified among scions, but very few were detected among rootstocks (Fig. S1). Genetic diversity indices are summarized for each locus (Table S2). The total number of alleles for each locus ranged from 3 (JR09 and JS28) to 12 (JS12) with an average value of 6.74, while the mean observed heterozygosity (*H*_O_) and expected heterozygosity (*H*_E_) were 0.58 and 0.56, respectively. Mean effective number of alleles (*N*_E_), Shannon’s information index (*I*), and unbiased expected heterozygosity (*uH*_E_) among loci were 2.53, 1.06, and 0.56, respectively.

**Table 1:** Sampling information of *J. sigillata* grafting system collected in Yunnan, China. The suffix of S and R represent scion and rootstock, respectively

Population ID	Sample size	Prefecture	County	Location	Longitude (E)	Latitude (N)	Elevation (m)
LH-S/LH-R	6/6	Nujiang	Lanping	Hexi Xiang	99.3819	26.8642	2179
YS-S/YS-R	20/20	Lijiang	Yulong	Shigu Town	99.9309	26.8608	1849
YG-S/YG-R	20/20	Dali	Yunlong	Gongguo Town	99.2833	25.6034	1470
YB-S/YB-R	20/20	Dali	Yongping	Taiping Xiang	99.8452	25.6044	2000
GY-S/GY-R	19/19	Dali	Yongping	Changjie Xiang	99.705	25.2983	2147
GB-S/GB-R	4/4	Baoshan	Longyang	Mangkuan Xiang	98.7898	25.2964	1886
CN-S/CN-R	22/22	Baoshan	Changning	Goujie Xiang	99.8499	25.0324	1832
FQ-S/FQ-R	19/19	Lincang	Fengqing	Xiaowan Town	100.0239	24.7109	2035
YD-S/YD-R	19/19	Lincang	Yongde	Yalian Xiang	99.5909	24.2269	1658

Three datasets were compiled, comprising only scions (S), only rootstocks (R), and all samples (T). For each of the  datasets and populations, genetic diversity parameters were calculated across loci (Table 2). A total of 209 alleles were identified for all samples, with the rootstocks exhibiting a higher total number of alleles (*N*_T_) (total of 194, and an average of 110 per population) than the scions (total of 171 with an average of 88 per population). The number of private alleles (*N*_P_) was higher in the rootstocks (28) than among the scions (13). Similarly, other genetic diversity parameters, namely mean number of alleles (*N*_A_) (5.52/6.26), effective number of alleles (*N*_E_) (2.25/2.65), *H*_E_ (0.51/0.58) and allelic richness (*A*_R_) (5.55/6.45) were lower in scions than in rootstocks. However, scions had a slightly higher observed heterozygosity (*H*_O_ = 0.63) than rootstocks (0.53). At the population level (except for populations LH and YS), all genetic diversity indices other than *H*_O_ were higher in rootstock than in scions. All loci were polymorphic within each dataset (T, S, and R), but the proportion of polymorphic loci (*PPL*) ranged from 51.6% to 100% for populations. Seven scion populations had negative *F*_IS_ values, while this was the case for only two of the rootstock populations. Finally, both the Wilcoxon and standardized difference tests independently indicated that two scion populations (YB-S and GB-S) had experienced significant bottlenecks (*P* < 0.05), whereas an additional four scion (LH-S, YB-S, GB-S and CN-S) and two rootstock (YS-R and CN-R) populations showed a bottleneck signal in one test but not the other (Table 2).

**Table 2:** Genetic diversity indices and bottleneck test results for whole dataset (T), Scions (S) and Rootstocks (R), and per population of scion and rootstock

Dataset & Population ID	*N*_T_	*N*_P_	*N*_A_	*N*_E_	*H*_O_	*H*_E_	*A*_R_	*PPL *(%)	*F*_IS_	TPM	
										Standardized	Wilcoxon test
T	209	-	6.74	2.53	0.58	0.56	6.97	100	-0.036	**-**	**-**
S/R	171/194	13/28	5.52/6.26	2.25/2.65	0.63/0.53	0.51/0.58	5.55/6.45	100/100	-0.236/0.083	**-**	**-**
LH-S/LH-R	110/101	1/2	3.55/3.26	2.71/2.49	0.53/0.54	0.58/0.54	3.25/3.12	96.8/93.6	0.172/0.086	0.068/0.070	0.036*/0.051
YS-S/YS-R	127/110	4/1	4.10/3.55	2.73/2.49	0.59/0.56	0.58/0.54	3.15/2.85	100/96.8	0.010/-0.010	0.151/0.047*	0.281/0.077
YG-S/YG-R	92/122	3/5	2.97/3.94	1.95/2.46	0.56/0.53	0.44/0.55	2.32/2.95	100/100	-0.254/0.062	0.400/0.323	0.480/0.492
YB-S/YB-R	73/120	1/3	2.35/3.87	1.77/2.32	0.70/0.55	0.38/0.52	1.86/2.84	87.1/100	-0.840/-0.037	0.009*/0.176	0.010*/0.357
GY-S/GY-R	79/109	0/3	2.55/3.52	1.83/2.13	0.68/0.50	0.40/0.49	1.98/2.69	90.3/100	-0.701/0.001	0.106/0.122	0.186/0.504
GB-S/GB-R	50/72	1/0	1.61/2.32	1.57/1.85	0.52/0.42	0.27/0.41	1.68/2.42	51.6/90.3	-0.910/0.119	0.000*/0.342	0.000*/0.711
CN-S/CN-R	80/131	0/9	2.58/4.23	1.89/2.47	0.73/0.56	0.42/0.55	2.07/2.93	100/100	-0.716/0.006	0.120/0.048*	0.039*/0.421
FQ-S/FQ-R	85/108	3/4	2.74/3.48	1.93/2.20	0.57/0.50	0.41/0.49	2.30/2.73	87.1/100	-0.344/0.025	0.120/0.439	0.129/0.399
YD-S/YD-R	94/118	0/1	3.03/3.81	1.99/2.28	0.66/0.52	0.45/0.53	2.26/2.83	100/100	-0.427/0.041	0.385/0.261	0.542/0.692
Mean	88/110	1.4/3.1	2.83/3.55	2.04/2.30	0.61/0.52	0.44/0.51	2.32/2.82	90.3/97.9	-0.446/0.033	-	-

*Note*: *N*_T_ total number of alleles, *N*_P_ private alleles, *N*_A_ mean number of alleles, *N*_E_ effective number of alleles, *H*_O_ observed heterozygosity, *H*_E_ expected heterozygosity, *A*_R_ allelic richness, *PPL* percentage of polymorphic loci, *F*_IS_ inbreeding coefficient, *TPM* two-phase mutation model, populations that experienced recent bottlenecks (*P*<0.05) are marked with an asterisk (*). Those populations where a bottleneck signal was detected by both approaches are in bold. “–” indicates not calculated

### Patterns of genetic differentiation and population structure

**Fig. 3:**
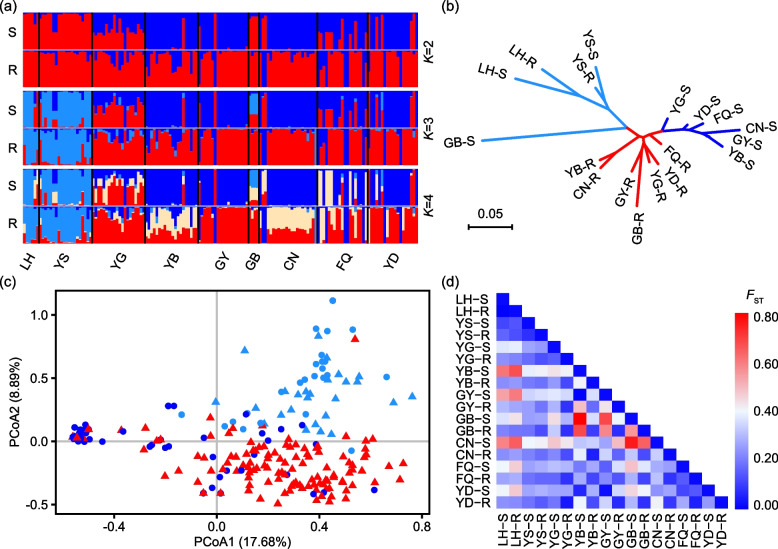
Genetic relationship and differentiation among scions and rootstocks of *Juglans sigillata* from nine sites. **a** Bayesian clustering of 149 scions (S) and 149 rootstocks (R) from 9 sites of *J. sigillata* from *K* = 2 to *K* = 4. **b** A radial distance neighbor-joining (NJ) tree showing relationships among all nine population pairs of scions (S) and rootstocks (R). **c** Principal Coordinates Analysis (PCoA) of all rootstocks and scions. The first and second components explain 17.68% and 8.89% of variation, respectively. Dots and triangles denote scions and rootstocks respectively. **d** Heat map depicting pairwise *F*_ST_ for both scion and rootstock populations; the magnitude of differentiation is indicated on the color ramp ranging from 0.00 to 0.80

According to AMOVA, when all scion and rootstock populations were placed in a single dataset (T), the among-population and within-population genetic variation for the whole dataset was 26% and 74% respectively (Table 3). When the scion and rootstock populations were analysed separately, the scions (dataset S) showed higher among-population genetic variation (35%) than the rootstocks (dataset R; 14%). Among the three datasets, the populations of scions show the highest genetic differentiation (*F*_ST_ = 0.350), then rootstocks (*F*_ST_ = 0.136), then all populations together (*F*_ST_ = 0.259) (Table 3). The analysis revealed a moderate genetic differentiation between scions and rootstocks (*F*_ST_ = 0.115). Generally, we found a low to moderate pairwise genetic differentiation (*F*_ST_) between all pairs of sampled populations with LH-R showing genetic distinctiveness from the rest of the populations of both rootstocks and scions (Fig. 3d; Table S3).

**Table 3:** Analysis of molecular variance (AMOVA) quantifying the distribution of genetic variation for three datasets of *Juglans sigillata*. Dataset T = all populations together, S = scions only, and R = rootstocks only

Datasets	Source	d.f	SS	MS	Percentage of variation
T	Among Pops	17	1449.24	85.25	26%
	Within Pops	280	3548.67	12.67	74%
	Total	297	4997.91		100%
	*F*_ST_	0.259			
S	Among Pops	8	642.78	80.35	35%
	Within Pops	140	1153.66	8.24	65%
	Total	148	1796.44		100%
	*F*_ST_	0.350			
R	Among Pops	8	486.10	60.76	14%
	Within Pops	140	2395.01	17.11	86%
	Total	148	2881.11		100%
	*F*_ST_	0.136			

*Pops* Populations, *d.f.* Degree of freedom, *SS* Sum of the squares, *MS* Mean sum of squares

The STRUCTURE analysis (Fig. 3a) revealed that *ΔK* had its maximum value at *K* = 2, making this the optimal *K* value (Fig. S2). At *K* = 2, one cluster contains mainly scions and the other mostly rootstocks, except for three scion populations (LH-S, YS-S and GB-S) which grouped with the rootstocks. Three other scion populations, YG-S, FQ-S, and YD-S showed admixture between the two clusters. Most rootstock populations other than LH-R and GY-R showed some degree of admixture within populations, often with certain individuals belonging to the scion cluster. When *K* = 3, a third genetic cluster is formed containing both scions and rootstocks from populations LH and YS, plus scions of GB-S. At *K* = 4, another new admixed genetic cluster emerges containing mainly parts of individuals in YB-R, CN-R, YG-S and GB-S, plus some individuals of FQ-S.

The radial neighbor-joining (NJ) tree divided the 18 populations into three major groups (Fig. 3b). Of these, one comprised scions only (six populations, i.e. YG-S, YD-S, FQ-S, CN-S, GY-S and YB-S), the second comprised rootstocks only (seven populations, i.e. YB-R, CN-R, GY-R, GB-R, YG-R, YD-R, and FQ-R), while the third cluster comprised both scion and rootstock population of YS and LH, plus GB-S. This clustering pattern was generally consistent with that observed in STRUCTURE at *K* = 3 (Fig. 3a).

PCoA results indicated that most scions clustered closely, with a few mixing with rootstocks. Most rootstocks showed a much more dispersed distribution than scions (Fig. 3c). These three groupings were also generally consistent with STRUCTURE results at *K* = 3 (Fig. 3a).

### Spatial genetic structure

**Fig. 4:**
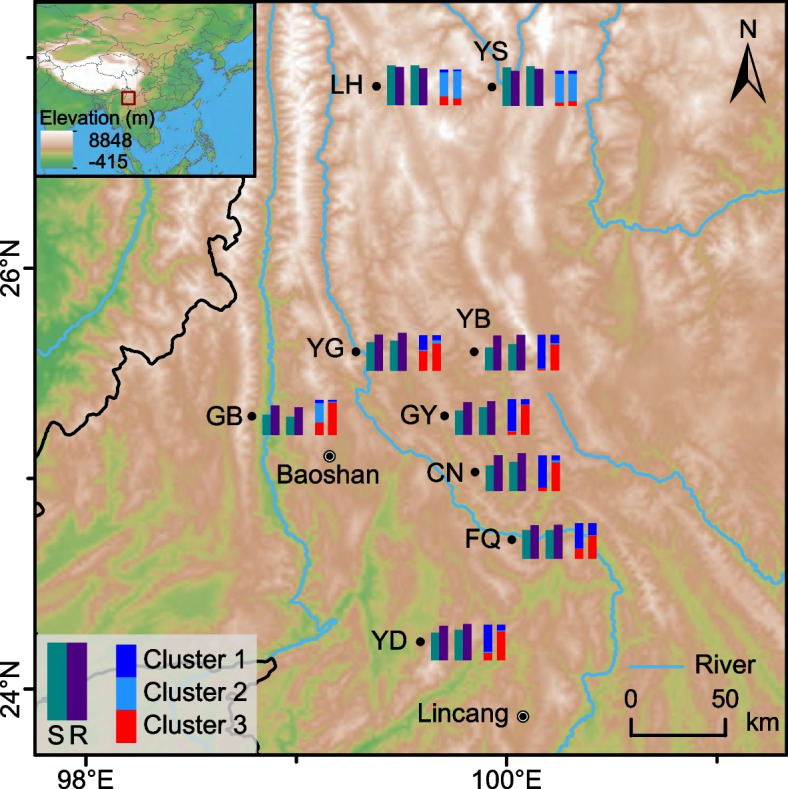
Spatial distribution of genetic diversity of the scions and rootstocks of iron walnut in Yunnan Province. The left-upper inset shows the geographical location of the study region in Asia. The dark points indicate sampling localities along with their population codes (see Table 1). For each site, the histogram represents the *A*_R_, *H*_E_ and genetic structure (*K* = 3) for scion (S) and rootstock (R) from left to right. The genetic proportions of each cluster correspond to those in Fig. 3a

Due to the high positive association among the various genetic diversity indices (Table 2), we mapped only the geographical distribution of two genetic diversity index (*A*_R_ and *H*_E_) and genetic structure (at *K* = 3) (Fig. 4). The results suggest that for the seven sites in the southern region (i.e. YD, FQ, CN, GY, YB, YG, and GB), the genetic diversity of scions was lower than the value for rootstocks. However, an opposite trend was revealed in two northern sites (YS and LH). Regarding genetic structure, the genetic composition of six sites (i.e. YD, FQ, CN, GY, YB, and YG) from the southern region showed clear genetic differentiation between scions and rootstocks, but with some genetic overlap between them. The two northern sites showed similar genetic composition to one another, but within them, no obvious genetic differences were observed between scions and rootstocks. Population GB had a striking genetic difference between scions and rootstocks, with the scions (GB-S) being more similar to the scions of two northern sites, whereas the rootstocks (GB-R) were closest to the rootstocks from the rest of the southern populations (Figs. 3 and 4).

## Discussion

Natural forests of *J. sigillata* provide a reservoir of genetic diversity, which is potentially under threat from the use of wild trees as rootstocks to graft cultivars for walnut industry. Therefore, the iron walnut is an ideal model to examine how grafting affects genetic diversity and population structure in fruit crops, and possibly other wild plants.

### Erosion of genetic diversity induced by grafting

The levels of genetic diversity in a plant species are often determined by intrinsic (e.g. life form, population history, and breeding system) and extrinsic (e.g. ecological factors, interspecific competition and human activity) factors [3, 4]. Globally, there has been an exceptionally rapid loss of genetic diversity due to intensive anthropogenically-driven environmental change [32, 33]. One such anthropogenic activity, the grafting of a few agronomically elite cultivars onto natural trees, may cause rapid genetic diversity loss (Fig. 1). Each such graft removes from the population one naturally sown tree, and hence reduces natural genetic diversity. Our results do indeed show that genetic diversity is being eroded by grafting within natural populations, because at least 23 alleles (*N*_T_) including 15 private alleles (*N*_P_) have been lost in the scions relative to the rootstocks, and all genetic diversity indices except *H*_O_ are lower in scions than rootstocks (Table 2). The higher *H*_O_ in scions indicates heterozygote excess, which is expected because recurrent artificial clonal propagation tends to preserve highly fit genotypes exhibiting heterosis and has also been observed in clonal material of *Prunus amygdala* “Halwani” [34] and *P. avium* [10]. At the population level, the same pattern of scions having lower diversity than rootstocks was repeated in all populations except the northern populations LH and YS (Fig. 4; Table 2; see below second point for more discussion).

The levels of genetic diversity observed within the rootstock populations (Table 2) were generally comparable to that of *Citrus* rootstocks in Tunisia [35], but lower than that for suckers derived from *Olea europaea* rootstocks in the Mediterranean [27] and even the seed propagated ‘Khachabi’ cultivar of *Prunus amygdalus* in Lebanon [34]. However, such direct comparison of genetic diversity among species should be done cautiously due to variations in life history traits, molecular markers and even the sources of rootstocks. The relatively low levels of genetic diversity of rootstocks in the current study may indicate that certain wild populations of *J. sigillata* may already contain rootstocks originating from naturalized cultivars. Consistent with this, the common walnut (*J. regia*) exists as naturalized rather than native populations in most of Europe [36] and China [37]. Therefore, a similar scenario might be true for *J. sigillata*. The genetic diversity among *J. sigillata* scions (Table 2) is also lower than that for Mediterranean *Olea europaea* scions [27], but is consistent with the generally low genetic diversity of *J*. *sigillata* cultivars from southwest China [28]. Such patterns clearly reflect both the narrow genetic background of cultivars, and the tendency to clonally propagate a few choice genotypes, for example by grafting [28]. One cause of low genetic diversity in cultivars is bottleneck events (Table 2) during selective breeding, as previously seen in *Prunus avium* [38].

### Distortion of population structure in grafted populations

Our results revealed a moderate genetic differentiation between scions and rootstocks (*F*_ST_ = 0.115; Fig. 1), and hence grafting may have caused changes to population genetic structure in natural walnut forests. Clear genetic divergence between scion and rootstock populations was observed in clustering analysis for the seven sites in the southern region (Figs. 3 and 4; Table S1). No fertile shoots were observed from below the grafting mark on any sampled individual, indicating that population structure of wild iron walnut may have been permanently distorted by grafting. Moreover, there was considerable genetic admixture between scions and rootstocks (Fig. 3), which might suggest crop-to-wild gene flow via seed or pollen from scions. Interfertility between *J. sigillata* and *J. regia* [25, 39, 40] points to the absence of breeding barriers within *J. sigillata*. Therefore, our data indicate that wild populations of *J. sigillata*, as represented by rootstocks examined here, have already been influenced by gene flow from cultivars, and that this trend is likely to increase with the rapid expansion of walnut plantations.

The low genetic differentiation between scions and rootstocks of two northern sites (LH and YS) (Fig. 4) and between the two sites (Fig. 3), suggested a genetic similarity between grafted walnut trees at these two sites, possibly indicating a common origin. Hence both scions and rootstocks in these populations appear to share a common origin distinct from other populations, and perhaps some or all of the scions might have been derived from genetically superior wild individuals or their cultivated descendants from those same populations. An alternative but yet to be verified explanation is the possibility of a cryptic genetic contribution from more northern *J. regia* populations to populations LH and YS. However, quantification of such gene flow may need broader geographical sampling.

When interpreting unexpected genetic similarity between rootstocks and scions, as observed in the northern sites LH and YS (Figs. 3 and 4; Table S3), the possibility of mislabeling or misidentification must be considered and, if possible, such similarity be ignored. The mislabeling problem was also encountered in the analysis of olive trees from the Mediterranean [27], mango germplasm evaluation [41], and walnut cultivar fingerprinting [28]. However, in the current study, only trees with clear grafting marks were sampled, and the knowledge of local people was further used to confirm that each sampled individual had been grafted. Moreover, great care was taken to sample scions and rootstocks above and below the mark, respectively (Figs. 1, 2). Therefore, mislabeling can be ruled out, and instead the scion/rootstock similarity in northern Yunnan likely results from a long cultivation history in the region, and we postulate that either the cultivars here were derived from local wild material independently from this process in the south, or rootstock populations in the north in fact derive from feralised local cultivars. Both hypotheses might be true to a degree, although during our fieldwork we only encountered what appeared to be fully wild populations of *J. sigillata* in the south; the two northern populations LH and YS grew near farmlands, and appeared to be semi-wild. This observation tentatively supports the hypothesis of northern rootstocks deriving from feralised cultivars (see above first point), providing a handy local grafting resource for farmers, and explaining the genetic similarity of rootstocks to scions in these populations.

Throughout Yunnan, we detected a relatively low genetic differentiation among rootstock populations, but a relatively high level of genetic differentiation among scion populations (Figs. 3 and 4; Table 3). The former is a common scenario for most outcrossing species [8], which *J. sigillata* is [26], and so is an expected result for rootstocks, which originate as individuals within a wild population. Conversely, the relatively higher genetic divergence among scion populations is likely an artificial factor, reflecting the diverse origins of cultivars used for scions. Compared to rootstocks, less gene flow is expected among scions due to asexual propagation, and in fact, local adaptation and artificial selection would generate gradual divergence between cultivar populations used as scions. However, artificial movement of scion material between populations would enhance variation within populations while reducing differentiation between them, much like gene flow in natural populations. Such movement might, for instance, explain the strong similarity between populations LH and YS, or the scions of CN, GY and YB (Figs. 3 and 4). Overall, our research indicates that most scions form a distinct genetic population of cultivars that is distinct from the rootstocks, but that northern cultivars might have a separate origin, and/or that northern rootstocks represent naturalized rather than native material. Meanwhile, the question of the origin of cultivated iron walnut remains unresolved and needs more taxonomic, geographical, and genomic sampling in the future.

### Implications for conservation and crop improvement

Genetic diversity is the fundamental basis for population fitness and maintenance of adaptive potential to respond to environmental changes, and hence has great conservation significance [1, 2, 42]. The extant genetic diversity of a species is a product of mutation, genetic drift, and selection over a long period [4, 43], but can be rapidly reduced by anthropogenic activities. Our data confirm that the replacement of wild individuals by grafted scions can have such an effect in *J. sigillata,* wherein scions exhibit lower genetic diversity than rootstocks (Fig. 4; Table 2), a scenario that might also be present in other crop species, e.g., grafted olive trees [27]. Loss of genetic diversity among scions might result in reduced ability to withstand biotic factors such as pests and pathogens, which are currently facing the walnut industry. Likewise, reduced genetic diversity in wild rootstock populations reduces the available natural genetic variation that could be used to buffer against such threats. These findings hence indicate that genetic diversity may be eroded from natural forests if large numbers of wild individuals are replaced by a few grafted cultivars. Therefore, in situ conservation of intact wild trees is recommended, because wild populations that retain genetic diversity can preserve co-adapted gene complexes as well as alleles not present in cultivated material, and hence have the capacity to evolve resilience in the wake of novel abiotic and biotic stresses [2]. With regard to iron walnut, we recommend that the remnant wild forest that includes the seven southern sites (Fig. 4) should be prioritised for in situ conservation. In addition, we recommend adoption of ex situ conservation, especially for material from sites with multiple private alleles. Since rootstocks used as grafting material no longer produce seeds (Fig. 2), seed collection from these rootstocks should focus on the remnant mature iron walnut trees. However, in populations where few or no such individuals remain, there might be a possibility to propagate wild material via offshoots from rootstocks. Moreover, breeding among ex situ populations should be done cautiously, because highly structured populations are prone to outbreeding depression [43, 44]. Finally, to ensure long term preservation and maintenance of genetic diversity, and hence overall crop health, we recommend that farmers adopt a mixed plantation approach encompassing both seed-based propagation of wild trees and the grafting of cultivars of iron walnut away from wild material.

## Conclusions

Detailed knowledge regarding the genetic diversity and differentiation of scions and rootstocks of a species provides important information for the conservation of genetic resources to prevent future loss of genetic diversity caused by global environmental change. Our results indicated that rootstocks had higher levels of genetic diversity than scions, suggesting that grafting onto wild trees causes genetic erosion. Meanwhile, contrasting genetic composition between scions and rootstocks, hinted that the population structure was distorted due to grafting. Therefore, conservation action is urgently needed, and we recommend both ex situ and in situ conservation of natural *J*. *sigillata* populations*,* and caution against the overuse of grafting onto natural rootstocks. The same issues are likely to occur in other crop trees where wild trees are used as grafting for scions, in Yunnan (e.g. pear and persimmon) and elsewhere. For future studies, we recommend inclusion of range-wide sampling with genomic, phenotypic and environmental data, to examine differences between rootstocks and scions and examine the impact of grafting upon adaptability to stresses such as pathogens and climate change.

## Methods

### Population sampling

Between 2016 and 2018, a total of 149 grafted trees were sampled from nine sites containing natural *J*. *sigillata* populations that include grafted individuals, distributed across the five well known walnut production areas of Yunnan Province (Table 1). We sampled 19 to 22 individuals at all sites except LH and GB, where only 6 and 4 individuals could be sampled, respectively, due to annual intensive management that had removed all new shoots from most rootstocks. All sampled populations occupied semi-natural habitats that are currently facing considerable anthropogenic pressure (Fig. 2). To avoid sampling natural clones, all sampled trees were separated by at least 50 m. Based on information gathered in the field, all sampled individuals were first identified as having been grafted within the past 30 years. Grafted trees were identified by the presence of clear grafting marks separating the trunk from the shoot (Figs. 1 and 2), and confirmed by interviewing local people. From each individual, samples were collected from above and below the grafting mark separately, with the former sampling the scion and the latter the rootstock (Fig. 2d, e). Only clean and healthy leaves were sampled and subsequently dried in silica gel. Dr. Jie Liu identified voucher specimens, and further prepared and deposited them in the Herbarium of Kunming Institute of Botany, Chinese Academy of Sciences (KUN).

### DNA extraction and SSR genotyping

Total genomic DNA was extracted from about 20 mg of silica gel-dried leaf tissue following a modified CTAB procedure [45, 46], and the concentrations of DNA solutions were adjusted to 30–50 ng/µL for downstream PCR amplification. The 298 samples from 149 grafted trees were genotyped using 31 SSR primer pairs used previously in our studies. This set of polymorphic primers was previously developed using an approach that integrates genome screening and cross-species amplification [31]. Subsequently, these loci have proved to be reliable in characterizing the genetic diversity and population structure of walnut [28–30]. These microsatellite loci included 20 with tri, seven with tetra, three with penta, and one with hexa repeat motifs (Table S1). All the forward primers were fluorescently labeled with FAM, HEX, or TAMRA dyes (Optimus Bio, Kunming, China) at the 5’ end. The 31 primer pairs were grouped in five multiplexes based on size and color following our previous studies [28, 29]. Multiplex PCR amplification was conducted on a Veriti® 96-Well Thermo-Cycler (Applied Biosystems, Foster City, California, USA) using a 15 µL multiplex PCR composed of 1 µL each of reverse and forward primers, 2 µL of the DNA template, and the remaining volume topped up with Golden Star T6 Super PCR Mix (Tsingke, Beijing, China). The following cycling regime was used: initial denaturation at 98 °C for 2 min, 35 cycles at 98 °C for 10 s, primer annealing temperatures (53 °C -61 °C) (Table S1) for 15 s, 72 °C for 10 s, then a final extension at 72 °C for 5 min, with a holding temperature of 4 °C. The fragment sizes of PCR products were determined using an ABI 3730xl DNA Analyzer (Applied Biosystems, Foster City, CA, USA).

### Data organization

We used GENEMARKER v2.2.0 (SoftGenetics, State College, PA, USA) for data viewing and allele scoring. Null and stuttered alleles were examined by MICRO-CHECKER v2.2.3 [47], whereas GENODIVE v3.0 [48] was used to identify multi-locus genotypes (MLGs) for both the scions and rootstocks.

At the population level, scions and rootstocks at each site were treated as separate populations (e.g. CN-S and CN-R refer to the scion and rootstock populations from site CN (Goujie Xiang (耈街乡), Changning county (昌宁县)), respectively) (Tables 1 and 2). To allow for clear comparison and analysis, three datasets were compiled: dataset T comprised all populations, whereas datasets S and R comprised all scion, and all rootstock populations respectively.

### Estimation of genetic diversity

The level of polymorphism of microsatellite loci for the current study was tested by calculating the number of alleles (*N*_A_), effective number of alleles (*N*_E_), Shannon’s information index (*I*), observed (*H*_O_) and expected heterozygosity (*H*_E_), and unbiased expected (*uH*_E_) heterozygosity using GENALEX v6.51b2 [49]. For datasets T, S, and R, and each of the populations, the genetic diversity analyses were conducted in GENALEX for both the scion and rootstock populations. In addition to the diversity parameters estimated for SSR loci, other indices including the total number of alleles (*N*_T_), private alleles (*N*_P_), and percentage of polymorphic loci (*PPL*) were also calculated in GENALEX. Rarefied allelic richness (*A*_R_) was computed using *Hierfstat* R package [50], with a minimum sample size of four genotypes. Inbreeding coefficient (*F*_IS_) was calculated using FSTAT v2.9 [51]. To identify possible population bottlenecks based on the allele frequency spectrum, BOTTLENECK v1.2.02 [52] was used, applying the default settings and tests for microsatellite data (two-phased model: 90% one-step mutations and 30% multi-step changes, evaluation with standardized differences and Wilcoxon tests; [53]).

### Genetic differentiation and population structure analysis

The estimation of genetic differentiation was conducted for each of the three datasets. To minimize the impact of null alleles on this [54], estimation of pairwise differentiation (*F*_ST_) among populations involved a correction for null alleles using FreeNA [54], with a bootstrap resampling of 10 000, and results presented in the form of a heatmap using the *pheatmap* R package [55]. Analysis of molecular variance (AMOVA) was performed in GENALEX to quantify the partitioning of genetic variation both between and within populations. Additionally, a model-based clustering approach implemented in STRUCTURE v2.3.4 [56] was used to infer the clustering of individuals. This was run with different values of the numbers of clusters (*K*) varying from 1 to 15 under the admixture model with correlated allele frequencies and without a priori grouping assumptions. To confirm the reliability of the results, we performed 10 independent runs per *K* value with a 10 000 burn-in period and 100 000 MCMC replications. The best *K* value was then determined in the web-based STRUCTURE HARVESTER v0.6.94 [57]. Furthermore, principal coordinates analysis (PCoA) was used to infer genetic clustering among scions and rootstocks. The PCoA analysis was implemented in GENALEX with a standardized covariance matrix of genetic distances generated according to Smouse and Peakall [58]. Finally, an unrooted neighbor-joining tree (Nei’s genetic distance; 1 000 bootstrap replicates) was constructed using POPULATIONS v1.2.31 [59] and visualized with the online tool ITOL (https://itol.embl.de).

### Spatial distribution of genetic variation

To spatially link genetic diversity and genetic structure of scion and rootstock populations with geography, the genetic diversity indices (*A*_R_ and *H*_E_) and genetic structure (STRUCTURE result for *K* = 3) were subsequently mapped using ArcGIS v10.7 (ESRI, Redlands, CA, USA).

### Supplementary Information

**Additional file 1: Supplementary tables. Table S1.** Properties of 31 microsatellite loci used to characterize 298 walnut individuals. **Table S2.** Genetic diversity of the 31 microsatellite loci. **Table S3.** Population pairwise *F*_ST_ for both scion and rootstock populations. **Table S4.** The population and individual ID, voucher specimens and SSR genotyping data used in the current study.

**Additional file 2: Supplementary figures. Fig. S1.** Distribution of the pairwise number of allele differences among MLG (threshold=10): (a) Bimodal curve for scions, (b) Unimodal curve for rootstock genotypes. **Fig. S2. **Optimal *K* value of STRUCTURE selection graphs. (a) Delta (Δ)*K* for different numbers of subpopulations (*K*), maximum number of sub-populations were inferred at *K*=2 for STUCTURE analysis; (b) The average of log-likelihood value of *K*.

## Data Availability

All data in the study are included in this published article. Specifically, the genotyping data of SSR are available in Table S4.

## Abbreviations

SSRs: Simple sequence repeats
KUN: Herbarium of Kunming Institute of Botany, Chinese Academy of Sciences
*A*_R_: Allelic richness
*F*_IS_: Inbreeding coefficient
*F*_ST_: Genetic differentiation index
*H*_E_: Expected heterozygosity
*H*_O_: Observed heterozygosity
*I*: Shannon’s information index
*N*_A_: Number of alleles
*N*_E_: Effective number of alleles
*N*_P_: Private alleles
*N*_T_: Total number of alleles
*PPL*: Percentage of polymorphic loci
*uH*_E_: Unbiased expected heterozygosity
AMOVA: Analysis of molecular variance
PCoA: Principal coordinates analysis
NJ: Neighbor-joining
