# Supplementary material for: Genetic imprints of grafting in wild iron walnut populations in southwestern China
Source: BMC Plant Biol. 2023 Sep 13;23:423. doi: 10.1186/s12870-023-04428-z (PMC10498525; doi:10.1186/s12870-023-04428-z)
Supplement: Supplementary file 2 — Additional file 2: Supplementary figures. Fig. S1. Distribution of the pairwise number of allele differences among MLG (threshold=10): (a) Bimodal curve for scions, (b) Unimodal curve for rootstock genotypes. Fig. S2. Optimal K value of STRUCTURE selection graphs. (a) Delta (Δ)K for different numbers of subpopulations (K), maximum number of sub-populations were inferred at K=2 for STUCTURE analysis; (b) The average of log-likelihood value of K. [file 12870_2023_4428_MOESM2_ESM.docx]

**Additional file 2: Supplementary figures**


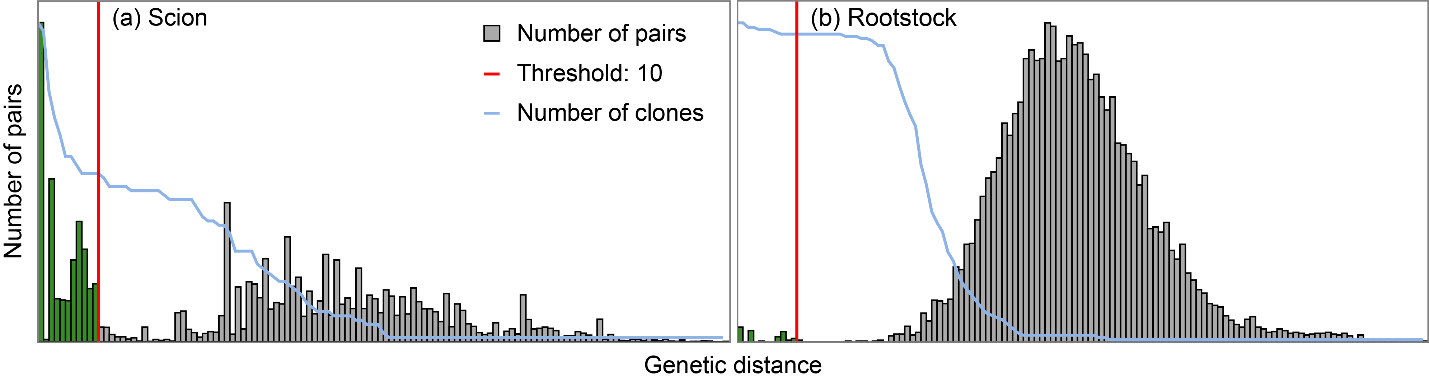


**Fig. S1** Distribution of the pairwise number of allele differences among MLG (threshold=10): (a) Bimodal curve for scions, (b) Unimodal curve for rootstock genotypes.


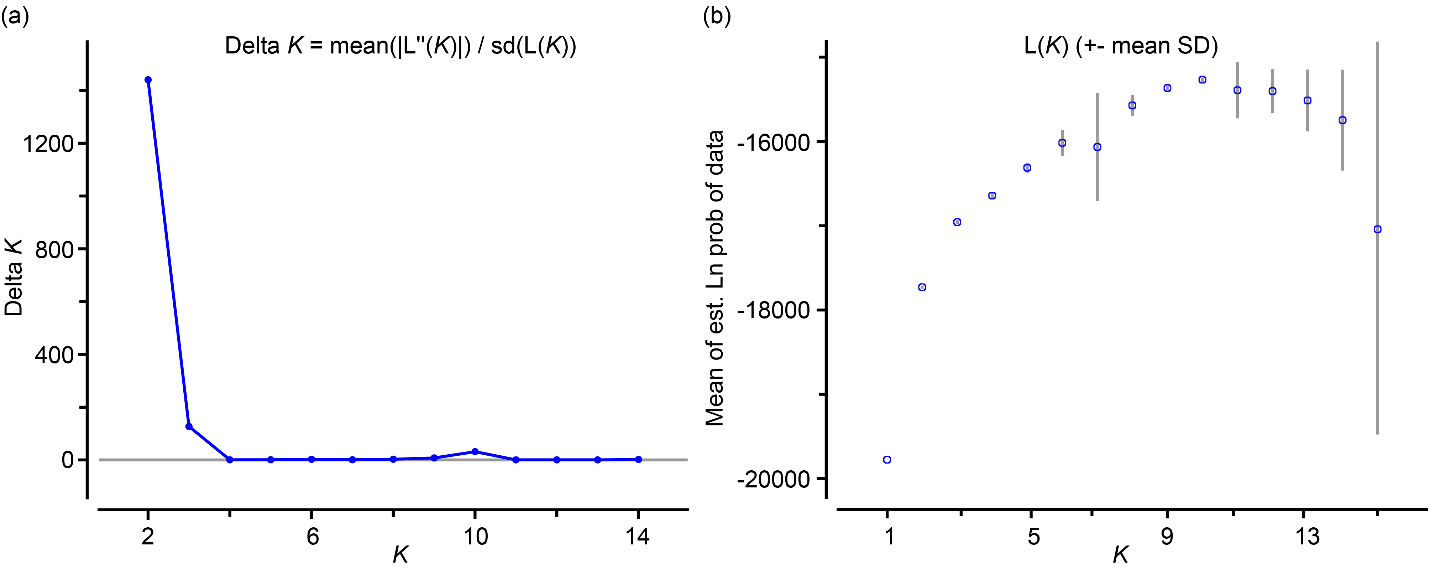


**Fig. S2.** Optimal *K* value of STRUCTURE selection graphs. (a) Delta (*Δ*)*K* for different numbers of subpopulations (*K*), maximum number of sub-populations were inferred at *K*=2 for STUCTURE analysis; (b) The average of log-likelihood value of *K*.
